# Higher Interrater Agreement of FDG-PET/CT than Bone Scintigraphy in Diagnosing Bone Recurrent Breast Cancer

**DOI:** 10.3390/diagnostics10121021

**Published:** 2020-11-28

**Authors:** Jorun Holm, Ziba Ahangarani Farahani, Oke Gerke, Christina Baun, Kirsten Falch, Hildebrandt Malene Grubbe

**Affiliations:** 1 Department of Nuclear Medicine, Odense University Hospital, 5000 Odense, Denmark; ziba.farahani2@rsyd.dk (Z.A.F.); Oke.Gerke@rsyd.dk (O.G.); Christina.Baun@rsyd.dk (C.B.); kirsten.falch@rsyd.dk (K.F.); Malene.Grubbe.Hildebrandt@rsyd.dk (M.G.H.); 2 Research Unit of Clinical Physiology and Nuclear Medicine, Department of Clinical research, University of Southern Denmark, 5000 Odense, Denmark; 3 Centre of Personalized Response Monitoring in Oncology (PREMIO), Odense University Hospital, 5000 Odense, Denmark; 4 Centre for Innovative Medical Technology (CIMT), Odense University Hospital, 5000 Odense, Denmark

**Keywords:** agreement, bone scintigraphy, breast cancer, interrater, positron emission tomography, recurrence, repeatability, reproducibility

## Abstract

The purpose was to investigate the interrater agreement of FDG-PET/CT and bone scintigraphy for diagnosing bone recurrence in breast cancer patients. A total of 100 women with suspected recurrence of breast cancer underwent planar whole-body bone scintigraphy with [99mTc]DPD and FDG-PET/CT. Scans were evaluated independently by experienced nuclear medicine physicians and the results for one modality were blinded to the other. Images were visually interpreted using a 4-point assessment scale (0 = no metastases, 1 = probably no metastases, 2 = probably metastases, 3 = definite metastases). Out of 100 women, 22 (22%) were verified with distant recurrence, 18 of these had bone involvement. The proportions of agreement between readers were 93% (86.3–96.6) for bone recurrence with FDG-PET/CT and 47% (37.5–56.7) for bone recurrence with planar bone scintigraphy. The strengths of agreement between readers for diagnosing bone recurrence was ‘almost perfect’ with FDG-PET/CT and was ‘fair’ with planar bone scintigraphy according to Cohen’s kappa value of 0.82 (0.70–0.95) and 0.28 (0.18–0.39), respectively. Interrater agreement yielded improved reproducibility with FDG-PET/CT versus with bone scintigraphy when diagnosing recurrence with bone metastasis in this patient cohort.

## 1. Introduction

Recent international guidelines recommend minimal imaging work-up for metastatic breast cancer to include imaging of the chest and abdomen, preferably with a computed tomography (CT) scan, and of bone [1]. It mentions that positron emission tomography (PET)/CT may be used instead of CT and bone scans, but this is not stated as a recommendation. Details on how to perform the bone scan are not mentioned: e.g., bone scan with planar imaging vs. single-photon emission computed tomography (SPECT), whether hybrid CT should be included, or if the more novel fluoride-PET scan is recommended. Former studies of 2-deoxy-2-[18F]fluoro-D-glucose (FDG) PET-CT for the diagnosis of breast cancer metastases have shown significantly higher accuracy of FDG-PET/CT than conventional imaging with contrast-enhanced CT (ceCT), ultrasound, and/or planar bone scintigraphy [2,3,4,5,6,7,8,9,10,11,12,13,14,15,16]. These studies are primarily based on retrospective accuracy studies that have methodological limitations, including lack of sufficient reference standard, lack of blinding, and limited or no interrater reliability or agreement reports. We have previously reported higher accuracy for FDG-PET/CT compared to conventional imaging of ceCT and planar bone scintigraphy for the diagnosis of distant recurrence of breast cancer in a prospective study using histopathology and follow-up as the reference standard [17]. However, we did not report on interrater agreement, and since we consider changing recommendations for the diagnostic workup for suspected metastatic breast cancer, this would affect a large group of patients, and analyses of reproducibility such as interrater agreement should be considered as recommended by the Guidelines for Reporting Reliability and Agreement Studies (GRRAS) [18]. The GRRAS guidelines were proposed to ensure how to report both reliability and agreement, suggesting a separate follow-up publication in all larger diagnostic accuracy studies. Clinical guidelines in oncology rarely mention reliability and agreement in imaging recommendations, and accuracy studies are often the basis for evaluating the level of evidence for a diagnostic modality. The GRRAS publication does not reflect on what influence reliability and agreement should play in evaluating the level of evidence for clinical guideline algorithms, but they do stress the need for rigorous studies on these matters in the future. The GRRAS guidelines were used in this brief article investigating interrater agreement between nuclear medicine experts in the data from our previous prospective study of FDG-PET/CT versus ceCT and planar bone scintigraphy in patients with clinically suspected recurrent breast cancer [17]. We aimed to investigate the interrater agreement for diagnosing bone recurrence when using FDG-PET/CT and comparing it with planar bone scintigraphy. We also investigated the interrater agreement for diagnosing distant recurrence, including extra-osseous metastases, using FDG-PET/CT.

## 2. Materials and Methods 

The study was conducted in compliance with the Declaration of Helsinki. Permission was given from the local ethics committee (S-20110138), approved on 28th of November 2011, and informed consent was obtained from all included patients.

The imaging data comprised the scans of women with suspected recurrence of breast cancer, performed at the Department of Nuclear Medicine in a prospective study at our institution from December 2011 to September 2014 [17]. The patients underwent planar whole-body bone scintigraphy with [99Tc]-dicarboxypropane diphosphonate and FDG-PET/CT within a median period of 3 days (range 0–24). Patients with suspected breast cancer recurrence or with verified local recurrence and potential distant metastases were invited to participate. Exclusion criteria were other malignancy, age less than 18 years, pregnancy or breast-feeding, diabetes mellitus, or considered unable to cooperate. The sample size calculation was based on accuracy considerations for the main study [17]: We assumed a prevalence of recurrence of 20% and based our calculation on a total sample size of 150 patients. The duration of the inclusion period was intended to be 2 years, but because of slower-than-expected recruitment, the number of patients included after 3 years was 102. The main reason for the closure of the study was that the time allotted had been exceeded. The reference standard was biopsy along with treatment decisions and clinical follow-up (median 19 months, range 1–35 months). In the current study, interrater agreement analyses of distant recurrence were performed for the FDG-PET/CT scan, and analyses of bone recurrence were compared with planar bone scintigraphy. Details for sample size calculations and scan procedures can be seen in the main publication [17]. 

### 2.1. Image Interpretation

One-hundred scans from the two imaging modalities were evaluated independently by experienced nuclear medicine physicians from our institution. The initial ratings for the main accuracy study were done by Z.A.F. (7-year experience) for the bone scans and M.G.H. (10-year experience) for the FDG-PET/CT scans. The subsequent ratings for the interrater study were done two years later, where the bone scans were evaluated by J.H. (7-year experience) and the FDG-PET/CT scans by Z.A.F. (9-year experience). All raters were aware of the referral text, but were blinded to each other, to any other imaging results, and other test results, e.g., the biopsy reports. Images were visually interpreted using a 4-point assessment scale: 0 = no sign of metastases, 1 = probably no metastases, 2 = probably metastases, and 3 = definite signs of metastases. Bone metastases were defined as metastases in the bone or bone marrow. Distant metastases were defined as all verified metastases other than local metastases in the operated breast and ipsilateral axilla; hence, distant metastases comprised metastases in all non-local regions, including bone metastases.

### 2.2. Statistical Analysis

Descriptive statistics were assessed according to data type: Continuous variables were displayed with medians and ranges, and categorical variables were characterized by frequencies that are identical with respective percentages due to a sample size of *n* = 100. Agreement analyses were based on proportions of agreement and Cohen’s kappa [19]. These estimates were supplemented by 95% confidence intervals (95% CIs) based on the Wilson-score method and bootstrapping techniques, respectively [20,21]. The level of significance was 5%, and the data were analyzed with Stata/MP 15 (StataCorp LP, College Station, TX 77845, USA). 

## 3. Results

Of the 102 patients who were initially included, one changed her mind, and another was excluded due to a previous known biopsy-verified bone metastasis, leaving 100 women available for analysis. Twenty-two patients out of 100 patients (22%) were diagnosed with distant recurrence, all diagnosed by biopsy; of those, 18 were classified as having bone metastases. Details on patient characteristics and other results can be seen in the main article [17], and additionally in Table 1.

### Interrater Agreement

**Figure 1:**
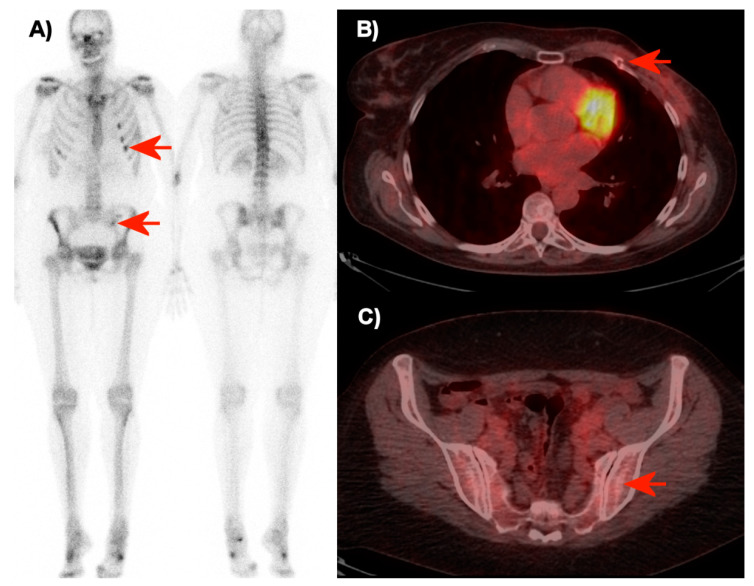
Bone scintigraphy (**A**) and transaxial FDG-PET/CT images over the thorax (**B**) and pelvis (**C**) of a 50-year-old woman without metastases (seven months of follow-up). The raters disagreed in the interpretation of the bone scintigraphy (**A**). Rater 2 interpreted these with point assessment 2 (probably metastases), while rater 3 interpreted the small focus in the left side of the pelvis and the successive foci in ribs (red arrows) with point assessment 1 (probably no metastases). On FDG-PET/CT (B + C), the two raters agreed about point assessment 0 (no metastasis). The focal FDG-uptake in the ribs was interpreted as sequelae of non-malignant traumatic fractures, and no focal FDG-uptake or structural pathology was observed in the left part of the pelvis on FDG-PET/CT (red arrows).

The proportion of agreement between readers was 80% (95% CI: 71.1–86.7) for diagnosing distant recurrence and 93% (95% CI: 86.3–96.6) for diagnosing bone recurrence with FDG-PET/CT. The proportion of agreement for diagnosing bone recurrence was 47% (95% CI: 37.5-56.7) with planar bone scintigraphy. The strengths of agreement between readers with FDG-PET/CT were ‘substantial’ for diagnosing distant recurrence and ‘almost perfect’ for diagnosing bone recurrence according to Cohen’s kappa values of 0.65 (0.52–0.78) and 0.82 (0.70–0.95), respectively. The agreement between readers for diagnosing bone recurrence with planar bone scintigraphy was ’fair’ with a Cohen’s kappa value of 0.28 (0.18–0.39). Cross tabulations of raters 1 and 2 for assessment of distant recurrence with FDG-PET/CT, bone recurrence with FDG-PET/CT, and bone recurrence with planar bone scintigraphy are shown in Table 2. Figure 1 is illustrating the interrater agreement with the interpretation of FDG-PET/CT versus the interrater non-agreement with the interpretation of the bone scintigraphy for one of the patients. 

## 4. Discussion

In this interrater agreement study, the proportions of agreement between readers were substantially higher with FDG-PET/CT than with planar bone scintigraphy for diagnosis of bone recurrence in breast cancer patients. Accuracy results have had the primary focus in the literature about diagnosis of recurrent breast cancer, and no previous reports of interrater agreement on recurrent breast cancer with FDG-PET/CT exist, to the best of our knowledge [11,12,13,14,17]. 

The strengths of this inter-rater agreement study are that it was performed prospectively, that histopathology was used as the reference standard, a short time (three days) between the imaging modalities, and interrater assessments were made by highly and rather equally experienced nuclear medicine specialists who were blinded to other test results. One of the raters (Z.A.F.) assessed both modalities, but with two years between the assessments, thus reducing the risk of a recall bias. The GRRAS guidelines suggest a clear statement of any crossing-over of raters or subjects in interrater studies to help readers decide whether the statistical analysis was appropriate. However, they do not state an optimal number of observers in interrater agreement studies.

Limitations of our study include the fact that it was performed only at a single institution, restricting generalization from our results. The three raters were employed in the same department and the agreement in readings of FDG-PET/CT may be more aligned as a result of similarity in background and training compared to a multicenter study. However, this alignment should apply to the inter-rater agreement for planar bone scintigraphy as well, which was not the case. Another limitation was that the power calculation was based on accuracy considerations for the main study alone. The sample size of 100 women is too small to extrapolate the results to other institutions or to propose alterations of clinical guidelines. We encourage more interrater studies in the future, because we find it clinically essential that the measurements of our diagnostic tools are of good quality regarding reproducible and reliability. The planar bone scintigraphy was performed without SPECT/CT, and the more novel bone modality sodium-[18F]fluoride PET/CT was not included for analysis. It could be expected that these modalities known to have better accuracy than planar bone scintigraphy would improve the interrater agreement for the bone scan, but we are not familiar with any interrater studies on bone recurrence in breast cancer performed with these modalities.

## 5. Conclusions

Interrater agreement was substantial for diagnosing distant recurrence with FDG-PET/CT, and it was significantly higher for FDG-PET/CT (almost perfect) than for planar bone scintigraphy (fair) when diagnosing bone recurrence. These results demonstrate an improved reproducibility of reporting FDG-PET/CT compared with planar bone scintigraphy in diagnosing bone recurrence in breast cancer patients. We suggest interrater agreement studies to become more integrated into the development of diagnostic algorithms and guidelines for diagnosing recurrent breast cancer to ensure quality in reproducibility and reliability.

## Figures and Tables

**Table 1:** Characteristics of 100 Danish patients with breast cancer.

Description		Descriptive Statistics
Primary site	Left	55
	Right	42
	Bilateral	3
Type of surgery	Breast-conserving	59
	Mastectomy	41
Adjuvant chemo- and/or radiotherapy	Yes	92
	No	8
Histology of the primary tumor	Invasive ductal carcinoma	73
	Invasive lobular carcinoma	13
	Ductal carcinoma in situ	5
	Other	8
	Missing	1
Stage of disease at diagnosis ^1^	I	16
	II	44
	III	23
	Missing	17
Estrogen receptor status	Positive	79
	Negative	15
	Missing	6
Progesterone receptor status	Positive	37
	Negative	57
	Missing	6
HER-2 status	Positive	13
	Negative	81
	Missing	6
Sentinel node procedure	Yes	54
	No	42
	Missing	4
Axillary dissection	Yes	69
	No	29
	Missing	2
Years since treatment for primary breast cancer		4 (0, 30)
Tumor size in millimeters; missing: *n* = 7		17 (5, 110)
Total number of lymph nodes removed; missing: *n* = 6		14 (1, 32)
Number of positive lymph nodes; missing: *n* = 5		1 (0, 23)

^1^ Stage of the disease was defined according to the Bloom–Richardson grading system.

**Table 2:** Cross tabulations of raters 1 and 2 for assessment of distant recurrence with FDG-PET/CT, bone recurrence with FDG-PET/CT, and of rater 2 and 3 for bone recurrence with planar bone scintigraphy.

**Distant recurrence assessed with PET**					
**Rater 1**	**Rater 2**				Total (%)
	0	1	2	3	
0	**53**	7	1	0	61
1	5	**4**	0	1	10
2	2	2	**1**	1	6
3	0	0	1	**22**	23
Total	60	13	3	24	100
**Bone recurrence assessed with PET**					
**Rater 1**	**Rater 2**				Total (%)
	0	1	2	3	
0	**73**	2	0	1	76
1	1	**2**	0	1	4
2	0	1	**0**	1	2
3	0	0	0	**18**	18
Total	74	5	0	21	100
**Bone recurrence assessed with BS**					
**Rater 3**	**Rater 2**				Total (%)
	0	1	2	3	
0	**27**	35	0	0	62
1	0	**11**	2	0	13
2	0	3	**4**	0	7
3	1	1	11	**5**	18
Total	28	50	17	5	100

BS: bone scintigraphy. FDG: 2-deoxy-2-[18F]fluoro-D-glucose. PET: positron emission tomography. Scale: 0 = no sign of metastases, 1 = probably no metastases, 2 = probably metastases, 3 = definite signs of metastases.
